# Novel 2-Thiazolylhydrazone with Druggable Properties for Antifungal Application

**DOI:** 10.3390/jof11010069

**Published:** 2025-01-16

**Authors:** Wallace Cordeiro de Morais, Gustavo Henrique Oliveira Costa, Vinícius Leal Pitcella, João Victor Vanolli Protti, Carolina Paula de Souza Moreira, José Eduardo Gonçalves, Susana Johann, Renata Barbosa de Oliveira

**Affiliations:** 1 Departamento de Produtos Farmacêuticos, Faculdade de Farmácia, Universidade Federal de Minas Gerais, Belo Horizonte 31270-901, MG, Brazil; wallacemorais2010@hotmail.com (W.C.d.M.); ghocosta93@gmail.com (G.H.O.C.); viniciuspitcella@gmail.com (V.L.P.); dugoncalvesj@gmail.com (J.E.G.); 2 Centro de Desenvolvimento Analítico Farmacêutico (CEDAFAR), Faculdade de Farmácia, Universidade Federal de Minas Gerais, Belo Horizonte 31270-901, MG, Brazil; 3 Departamento de Microbiologia, Instituto de Ciências Biológicas, Universidade Federal de Minas Gerais, Belo Horizonte 31270-901, MG, Brazil; joaovvanolli@gmail.com (J.V.V.P.); sjohann@icb.ufmg.br (S.J.); 4 Serviço de Desenvolvimento Tecnológico Farmacêutico, Diretoria de Pesquisa e Desenvolvimento, Fundação Ezequiel Dias (FUNED), Belo Horizonte 30510-010, MG, Brazil; carolina.moreira@funed.mg.gov.br

**Keywords:** fungal infections, thiazolyl hydrazone, antifungal agents, drug development

## Abstract

Fungal infections have become a growing concern in healthcare, particularly in immunocompromised individuals, with species like *Candida*, *Cryptococcus*, and *Sporothrix* posing significant challenges due to rising resistance and limited treatment options. In response, novel antifungal agents are being explored, including thiazolyl hydrazones. This study focuses on the development of a novel thiazolylhydrazone derivative, RW3. RW3 was synthesized to improve its water solubility and pharmacokinetic properties. The compound demonstrated a broad antifungal spectrum, particularly effective against *Cryptococcus neoformans* and *Candida auris*, with minimal irritant potential and low cytotoxicity. RW3 showed favorable solubility and high intestinal permeability, indicating potential for oral administration. The results suggest RW3 as a promising lead for further development as a therapeutic agent for systemic fungal infections. These findings underscore the importance of optimizing drug properties to enhance efficacy and safety profiles, opening the path for the development of innovative antifungal treatments.

## 1. Introduction

Over the past few years, fungal infections have emerged as a concern in the medical field, with several types gaining attention due to their rising prevalence, resistance to treatment, the cost and toxicity associated with the most commonly used antifungal agents, and the impact on vulnerable populations [1]. These infections, often caused by opportunistic fungi, can lead to serious health complications, particularly in individuals with weakened immune systems [2].

One such infection is sporotrichosis, caused by fungi belonging to the genus *Sporothrix*. This disease typically manifests as skin lesions that may eventually spread to lymph nodes. It is commonly associated with warm-blooded animals, including humans, dogs, and mainly cats. Sporotrichosis is of particular concern in tropical and subtropical regions [3].

Candidiasis is an infection resulting from yeast species within the *Candida* genus. The most common species responsible for infections is *Candida albicans*, even though other species, like *Candida glabrata* and *Candida tropicalis*, can also cause infections. Normally, *Candida* lives in the human body without causing harm. However, under certain conditions, such as changes in the body’s immune system, *Candida* can overgrow and lead to an infection [4]. Another rising fungal infection is *Candida auris*, a yeast resistant to multiple drugs that has emerged as a significant threat to the healthcare system. It can cause severe bloodstream infections, pneumonia, and infections in other parts of the body, often affecting critically ill or immunocompromised patients. The difficulty in diagnosing *Candida auris* and its resistance to antifungal drugs have made it a growing challenge for healthcare professionals [5].

Cryptococcosis is another significant fungal infection resulting from *Cryptococcus neoformans* and *Cryptococcus gattii*. This infection is most dangerous for people with compromised immune systems, such as those with HIV/AIDS. It primarily affects the lungs and can spread to the brain, leading to meningitis. The rise of cryptococcosis has been linked to environmental factors and the growing number of individuals living with HIV [6].

These fungal infections underscore the importance of ongoing research, awareness, and effective treatment strategies, particularly as they continue to pose challenges in healthcare settings. Improved diagnostic techniques, early detection, and the search for novel antifungal agents are crucial in managing these infections and preventing their spread [7].

Compounds synthesized by the research group, including those containing the thiazolyl hydrazone moiety, have demonstrated antifungal effects, making them promising leads for innovative drug candidates [8,9]. The high lipophilicity is one of the main limitations of this type of compound [9]. The optimization of their chemical structure by introducing polar and ionizable substituents led to the development of a new series with more favorable pharmacokinetic properties, including a better absorption profile via the oral route. *E*,*Z*−Ethyl 2-(2-(2-(4-hydroxybutan-2-ylidene)-hydrazono)thiazol-4-yl)acetate (RJ44) was identified as the most promising compound of this class due to its potent activity against various species of *Candida* and *Cryptococcus*, low cytotoxicity, and physicochemical properties suitable for clinical application [8].

Continuing the search for RJ44 analogues aiming to obtain compounds with even better physicochemical properties, we describe in this work the synthesis of a more hydrophilic thiazolyl hydrazone, as well as the evaluation of its antifungal activity, irritative potential, and determination of the cytotoxicity, solubility, and intestinal permeability.

## 2. Materials and Methods

### 2.1. Synthesis

The analytical reagents were obtained from commercial sources and used without purification. Thin-layer chromatography (TLC) was performed using pre-coated 0.5 mm glass plates with a suspension of 8 g of silica gel 60 containing 13% *w*/*w* CaSO_4_ (Sigma-Aldrich, St. Louis, MO, USA) in 19 mL of distilled water. The melting points (mp) were determined on a Microquimica MQAPF 301 apparatus. Nuclear magnetic resonance (NMR) spectra were recorded on a Bruker AVANCE III HD 400 MHz spectrometer (Billerica, MA, USA), using tetramethylsilane (TMS) as the internal. Chemical shifts are expressed in δ (ppm) scale, and J values are given in Hz, with the multiplicity of signals referred to as singlet (s), triplet (t), and quartet (q). The synthesis and structural elucidation of thiosemicarbazone has been previously described in the literature [8].

#### Synthesis of (E)-2-(2-(4-(2-Ethoxy-2-oxoethyl)thiazol-2-yl)hydrazineylidene)-Propanoic Acid (RW3)

Thiazolylhydrazone RW3 was obtained by an equimolar reaction of (2*E*)-2-[2-(aminothioxomethyl)hydrazinylidene]propanoic acid [8] (0.150 g, 0.93 mmol) and ethyl 4-chloroacetoacetate (126 µL, 0.93 mmol) dissolved in isopropyl alcohol (18 mL). The reaction mixture was kept under magnetic stirring at 80 °C for approximately 3 h. The reaction was monitored by thin-layer chromatography (TLC) (eluent: ethyl acetate and 5 drops of acetic acid; developer: iodine vapor). At the end of the reaction, the solvent was removed under reduced pressure, 18 mL of distilled water was added, and the contents of the flask were transferred to a separatory funnel. Then, the mixture was extracted with ethyl acetate (3 × 30 mL). The organic phase was dried over anhydrous sodium sulfate, filtered, and the solvent was removed under reduced pressure. The resulting product was washed with 7 mL of diethyl ether, yielding RW3 as a light yellow solid (0.134 g, 53%). M.p.: 164.2–167.7 °C; ^1^H NMR (400 MHz, DMSO-d6): δ 6.77 (1H, s), 4.08 (2H, q, J = 7.2 Hz), 3.63 (2H, s), 2.05 (3H, s), 1.19 (3H, t, J = 7.2 Hz); ^13^C NMR (100 MHz, DMSO-d6): δ 169.9, 168.4, 165.6, 138.9, 107.7, 60.37, 36.6, 14.1, 12.7.

### 2.2. In Vitro Antifungal Assay

Broth microdilution testing was conducted following the guidelines in the CLSI document M27-A3 (2008) with modifications [10]. Briefly, the following yeast were used in the present study: *C. albicans* SC5314, *C. tropicalis* ATCC 750, *Candidozyma* (*Candida*) *auris* COLO 01, *C. parapsilosis* ATCC 22019, *Pichia kudravzevii* (*C. krusei*) ATCC 20298, *C. gattii* ATCC 24065, *C. neoformans* ATCC 24067, and *S. brasiliensis* (189). All fungus strains were stored at a temperature of −80 °C. Before conducting the experiments, the strains were cultivated on Sabouraud dextrose agar (HiMedia, Mumbai, India) at 35 °C to ensure their purity and viability. The yeast phase of *S. brasiliensis* was grown on brain–heart infusion agar (BHI; Difco, Detroit, MI, USA) with two successive passages of 7 days at 35 °C and CO_2_ 5% [11]. The compounds were tested at concentrations of 0.125–250 µg/mL. For comparative antifungal controls, fluconazole (Sigma-Aldrich, St. Louis, MO, USA), itraconazole (Sigma-Aldrich, St. Louis, MO, USA), and caspofungine (Sigma-Aldrich, St. Louis, MO, USA) were included. The plates were incubated at 35 °C for 48 h for yeast and for 7 days for *S. brasiliensis* at 35 °C and CO_2_ 5%. The tests were performed in triplicate. The endpoints were determined visually by comparison with the endpoints of the drug-free growth-control well. The value of the minimum inhibitory concentration (MIC) was defined as the lowest extract concentration at which the well was optically clear and was expressed in µg/mL.

### 2.3. HET-CAM Assay

*Gallus gallus domesticus* eggs (n = 3) at the eighth day of embryonic development were incubated at 37 ± 2 °C and 50% humidity until the test. On the tenth day, the solutions of RW3 (100 µM), 1% DMSO in saline (diluent), 0.1 mol/L NaOH (positive control), and saline solution (negative control) were placed on the chorioallantoic membrane. Following the application of 300 µL of each solution, according to the German protocol [12], the changes in the membrane were recorded for 300 s, indicating the time of onset of each observed phenomenon (hemorrhage, lysis, and coagulation) and using the following formula to calculate the Irritation Score (IS) (Equation (1)). Each experiment was repeated two times.

IS = (301 − sH)/300 × 5 + (301 − sL)/300 × 7 + (301 − sC)/300 × 9,(1)

where H = hemorrhage, L—lysis, C = coagulation, and s = start second.

### 2.4. Saturation Shake-Flask Solubility Method

The equilibrium solubility of RW3 samples was assessed using the shake-flask method. The samples were dispersed in buffers described in the United States Pharmacopeia at pH values of 2.0, 4.5, and 6.8. For each reported solubility result, three independent shake-flask experiments were carried out in parallel. For each experiment, the solid sample was added carefully using a spatula to 2 mL of the aqueous buffer in a glass vial, while stirring, until a heterogeneous system (solid sample and liquid) was obtained. The solution containing solid excess of the sample was then capped, and stirred at 37 °C, 100 rpm, for 24 h in orbital shaker (Tecnal, Campinas, Brazil). An aliquot of 1 mL of the suspension was centrifuged, and the supernatant was then collected and filtered through a 0.2 µm PVDF filter. The resulting solution was then diluted 200× in the mobile phase, and the concentration of the sample in each aliquot was measured by the HPLC method. The RW3 concentrations were determined by injections of 10 µL of the samples via HPLC, using a Waters Alliance system (New Castle, DE, USA). Chromatographic separations were obtained using a PhenoSphere-NEXT^®^ 5 µm C18 column (150 mm; 4,6 mm; Phenomenex^®^, Torrance, CA, USA) at 40° C. The mobile phase (1 mL/min) was composed of phosphate buffer 20 mM pH 4.0 and acetonitrile (50:50 *v*/*v*). The wavelength was set at 308 nm. The HPLC method employed was evaluated for linearity, precision, accuracy, and selectivity.

### 2.5. In Vitro Caco-2 Cytotoxicity Determinations

In vitro cytotoxicity assays were carried out following the protocol outlined in the ISO 10993-5 guideline (ISO, 2022) [13]. The human colon carcinoma cell line, Caco-2 cells (from ATCC), were cultured in 96-well plates for 24 h at a seeding density of 2 × 104 cells/well. The RW3 compound was dissolved in DMEM without phenol to prepare samples of 10, 25, and 50 µg/mL concentrations. Six replicates of each concentration for RW3 were assessed. This range of concentrations was chosen for reasons associated with the subsequent permeability tests. The experiments began by replacing the culture medium in each well with 100 µL of sample or control solutions, followed by a 4 h incubation at 37 °C in a CO_2_ incubator. Then, the solutions in each well were aspirated, and the cells were further incubated for 2 h with 30 µL of MTT solution (5 mg/mL, Sigma–Aldrich Corp., St. Louis, MO, USA, in DMEM and PBS buffer, 9:1 *v*/*v*). Next, formazan crystals were solubilized with 70 µL of isopropyl alcohol acidified with hydrochloric acid and quantified spectrophotometrically at 570 and 690 nm (Spectra Fluor plate reader, Tecan, Grödig, Austria). Cell viability was calculated based on the measured absorbance relative to the absorbance of the cells exposed to the negative controls (DMEM), which represented 100% cell viability, and for the positive control, we employed DMSO 50% in DMEM, which represented 0% cell viability.

### 2.6. In Vitro Caco-2 Permeability Assay

The Caco-2 cells (from ATCC) were cultured in 12-well Transwell^®^ (Corning Incorporated, New York, NY, USA) insert filters for 21 days at a seeding density of 5 × 10^4^ cells/cm^2^ to achieve confluence and differentiation. The integrity of the monolayer was examined by measuring the transepithelial electrical resistance (TEER) with an epithelial voltammeter Millicell-ERS (Merck SA—German, Darmstadt, Germany). Only cell monolayers with a TEER above 300 × cm^2^ were used for the transport assays.

Transport experiments were conducted by adding 10 µg/mL RW3 in HBSS buffer at pH 6.8 to the apical compartments of the Transwell^®^ plates (Corning, Corning, NY, USA), while the basolateral buffer was maintained at pH 7.4. The plates were agitated in an orbital shaker at 37 °C (100 rpm). Samples (100 µL) were collected from both the basolateral and apical sides at 30, 60, 90, 120, and 180 min. The apparent permeability coefficients (Papp, cm/s) were calculated using the Equation (2):

Papp = (VR/A × C0)/(dC/dt)(2)

where VR is the volume of the receiver compartment (basolateral or apical), DQ/Dt is the linear appearance rate of the compound on the receiver chamber (in ng/cm^3^/s), A is the membrane surface area (cm^2^), and C0 is the initial concentration in the donor compartment (ng/cm^3^). This calculation requires that the sink conditions are fulfilled; therefore, only receiver concentrations below 10% of the donor concentration were employed in the calculations.

## 3. Results

### 3.1. Design and Synthesis of RW3

**Figure 1:**
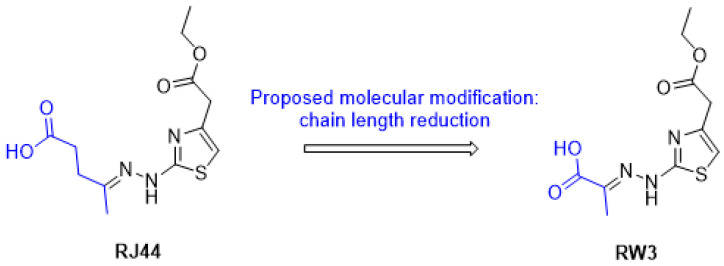
Strategy used in the design of a more hydrophilic thiazolylhydrazone based on the structure of the lead compound RJ44.

Based on the antifungal activity exhibited by RJ44 and with the aim of improving its water solubility, we proposed reducing the side chain of the hydrazone portion from five carbons to three carbons, while leaving the rest of the molecule unchanged, as shown in Figure 1. The reduction of the carbon chain is an efficient strategy for reducing the hydrophobicity of a molecule.

For comparison purposes, the logarithm of n-octanol/water partition coefficient (log Po/w) was predicted using the SwissADME web tool (http://www.swissadme.ch/index.php, accessed on 20 December 2024) [14], and, as expected, the logP value calculated for RW3 (logP = 1.18) was lower than the value calculated for RJ44 (logP = 1.57), indicating an increase in its hydrophilic character.

Considering the possible pharmacokinetic advantages of RW3 over RJ44, this compound was then synthesized in two steps from pyruvic acid and thiosemicarbazide using the methodology previously described [8].

### 3.2. Evaluation of the Antifungal Activity and Irritant Potential of RW3

Table 1 shows the Minimal Inhibitory Concentration (MIC) values of RW3 against various fungal species. The MIC is an important indicator of a compound’s effectiveness in inhibiting fungal growth, and the results highlight the varying efficacy of RW3 across different fungal species. The MIC values are expressed in micrograms per milliliter (µg/mL), and comparisons are made with the MIC values of standard antifungal agents like itraconazole, fluconazole, and caspofungin.

Additionally, to assess the possibility of irritation caused by RW3, the Hen’s Egg Test on the Chorioallantoic Membrane (HET-CAM) was performed. The HET-CAM assay is an in vivo semiquantitative test to evaluate the anti-inflammatory potential and anti-irritant properties, as well as the ocular toxicity of compounds/formulations. The benefits of this test include its simplicity, rapid execution, ease of use, and relatively low cost [15,16]. For the classification of the irritation potential (Table 2), the arithmetic mean of the six eggs was used, and the score was categorized as: non-irritant (0.0 to 0.9); mild irritant (1.0 to 4.9); moderate irritant (5.0 to 8.9) and severe irritant (9.0 to 21.0) [17].

**Figure 2:**
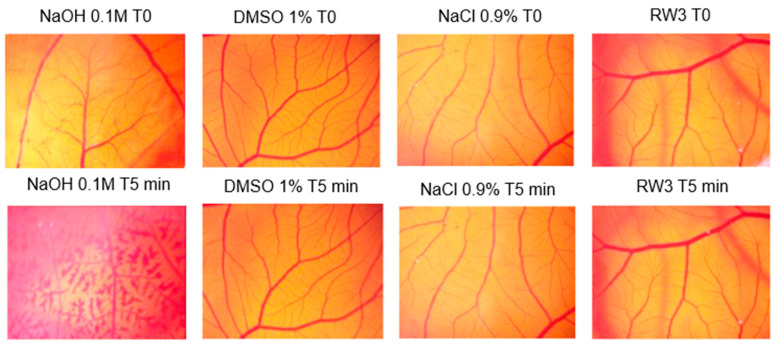
CAM images before sample addition (T0) and after 5 min of action (T5).

The CAM images at the beginning and end of the experiment are presented in Figure 2. It is possible to note the extensive hemorrhage caused by NaOH, the absence of irritation caused by the diluent and negative control (saline), and the little irritation caused by RW3.

### 3.3. Determination of Solubility of RW3

The determination of the solubility under physiological conditions, particularly considering the different pH levels throughout the gastrointestinal tract, is one of the most important biopharmaceutical parameters that must be generated during the early stages of developing a new drug for oral administration. In this study, the solubility determination of RW3 was carried out using the miniaturized shake flask test, as only a small amount of the compound under investigation was available. In the literature, there are publications that have successfully employed the miniaturized test [18,19].

The solubility results (S0) experimentally obtained for RW3 in buffers at pH 2.0, 4.5, and 6.8 are presented in Table 3. It was observed that RW3 exhibited increasing solubility with rising pH, in the order of 0.67 mg/mL, 1.01 mg/mL, and 2.76 mg/mL, at pH 2.0, 4.5, and 6.8, respectively.

According to the Biopharmaceutics Classification System (BCS) [20], a substance is classified as having high solubility when the ratio between the maximum daily administered dose and its intrinsic solubility (S0) under physiological pH conditions results in a value less than 250 mL, meaning that this dose can dissolve in 250 mL of aqueous medium.

The WHO, in its Biowaiver Guidance based on the Biopharmaceutics Classification System (BCS), states that when the solubilities of a substance differ across the three evaluated pH levels, the reference value for solubility classification should be the most critical value, i.e., the lowest solubility. In the case of RW3, this corresponds to the solubility at pH 2.0 [21].

### 3.4. In Vitro Cytotoxicity Determinations of RW3

**Figure 3:**
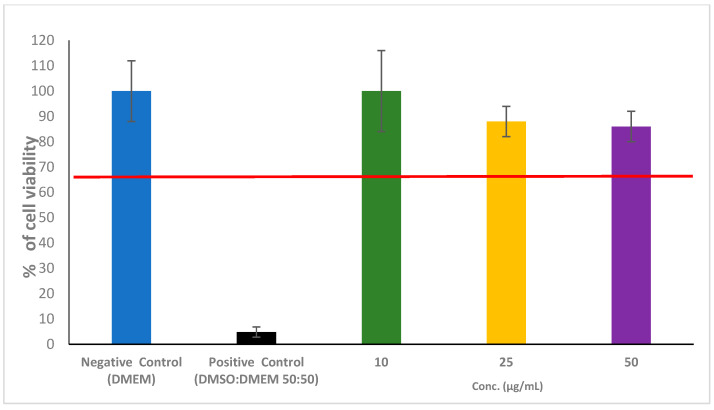
Viability of Caco-2 cells incubated for 4 h with RW3 assessed by the MTT technique. Each of the three concentrations was evaluated in six replicates. Error bars depict the primary results based on standard deviation. The red line separates the cell viability values above and below 70%.

The cytotoxicity of the RW3 in Caco-2 cells in vitro is shown as a percentage of the cell viability compared to the negative (100%) and positive controls, as measured by the MTT assay (Figure 3).

As shown in Figure 3, RW3 was tested at concentrations of 10, 25, and 50 µg/mL, resulting in cell viability rates of 100%, 88%, and 86%, respectively. According to the ISO 10993-5 standard, a material is non-cytotoxic, if the cell viability exceeds 70%.

### 3.5. Permeability Studies Using Caco-2 Cells

Human intestinal absorption of new drug candidates during the early stages of development is frequently evaluated by assessing the permeation of the drug across a monolayer of Caco-2 cells. Caco-2 cells are a well-established human epithelial colon carcinoma cell line that serves as a reliable in vitro model for studying intestinal drug absorption [22].

Permeability studies using Caco-2 cells are typically conducted in two primary directions: from the apical side (facing the intestinal lumen) to the basolateral (blood-facing) side, referred to as influx (A-B), and in the reverse direction, from basolateral to apical, termed efflux (B-A). The comparison of the apparent permeability coefficients (Papp) in these two directions provides critical insights into the involvement of active transport mechanisms, whether absorptive or secretory.

The Papp for RW3 were evaluated and are presented in Table 4. The value (Papp A-B = 23.08 × 10^−6^ cm/s) shown would be characterized as a high permeability compound (Papp > 10 × 10^−6^ cm/s) according to the Biopharmaceutical Classification System (SCB) [20,23,24].

The net flux ratio, which was lower than 2.0 (0.76) for the compounds, suggested that there was no significant transport in the efflux direction [22].

## 4. Discussion

The results of the antifungal activity evaluation show that RW3 demonstrates a wide range of activity against different *Candida* species. Among the *Candida* strains tested, *C. albicans* exhibited an MIC of 31.3 µg/mL, which is moderate when compared to the lower MIC observed for *C. auris* (15.6 µg/mL) and *P. kudravzevii* (15.6 µg/mL). In contrast, *C. tropicalis* and *C. parapsilosis* displayed a higher MIC of 125 µg/mL, indicating a lower sensitivity to RW3. These variations in MIC could be attributed to genetic differences among *Candida* species, affecting their susceptibility to antifungal agents.

Notably, the *Cryptococcus* species, *C. neoformans* and *C. gattii*, displayed relatively low MIC values of 3.9 µg/mL and 7.8 µg/mL, respectively. These results suggest that RW3 is particularly effective against *Cryptococcus* species, which is promising considering the clinical significance of *Cryptococcus* infections, especially in immunocompromised individuals. The lower MIC values for *Cryptococcus* species suggest a strong potential for RW3 as a therapeutic agent against these fungi.

RW3 showed an MIC of 15.6 µg/mL against *S. brasiliensis*, a significant pathogen in the *Sporothrix* complex, which is known to cause sporotrichosis. Compared to the MIC for itraconazole (0.5 µg/mL), RW3’s activity appears weaker against this species. However, it is still noteworthy that RW3 may offer an alternative treatment option, particularly in cases of resistance to conventional antifungal agents.

Regarding the irritant potential of RW3, it was observed that it is similar to that presented by the negative control (saline solution), that is, the compound has practically no potential for irritation. The HET-CAM assay is sensitive to evaluating agents and is well established for screening their ability to reduce inflammation [16,25].

Since RW3 is in the preclinical stage of development, nothing definitive can be stated regarding the potential therapeutic dose to classify it as having high or low solubility. However, by extrapolating a possible dose that would meet the criteria for high solubility classification, i.e., Class 1 of the Biopharmaceutics Classification System (BCS), a maximum daily dose of 168 mg can be considered as borderline. In other words, if the effective therapeutic daily dose required is less than 168 mg, the dose/S0 ratio would be less than 250, thereby classifying it as a high-solubility compound.

As shown in Figure 3, RW3 demonstrated no cytotoxic effects on the Caco-2 cell line at any of the tested concentrations. These results associated with the result of HET-CAM assay indicate that this compound can be considered safe due to its low toxicity.

Research that correlated the apparent permeability coefficient (Papp) with the percentage of human bioavailability (F%) suggests that compound RW3 is unlikely to encounter absorption issues in the gastrointestinal tract, provided it achieves adequate solubilization in physiological fluids. Research establishing a strong correlation between Papp values and human bioavailability has been conducted since the initial use of the model for predicting permeability [26]. Recently, new approaches have been employed to evaluate the predictive power of studies in cellular models and correlate these findings with computational models; we believe that the current in silico model is a dependable tool for identifying the most promising compounds with high intestinal permeability in the early stages of drug discovery and could be utilized in the development of provisional BCS classification [27,28].

## 5. Conclusions

In conclusion, RW3 shows promise as an effective antifungal agent, particularly against *Cryptococcus* species and *Candida auris*, with potential as an alternative treatment for antifungal-resistant infections. The compound exhibited favorable physicochemical properties, including improved water solubility, high intestinal permeability, and low cytotoxicity, suggesting its potential for oral administration. Additionally, RW3 demonstrated minimal irritant potential, highlighting its safety for further development. These findings indicate that RW3 could serve as a valuable potential treatment for systemic fungal infections, providing an alternative to traditional antifungal agents, particularly in light of increasing resistance. Future studies focusing on the optimization of its structure and clinical testing will be crucial to confirm its therapeutic potential and establish it as a viable treatment option in the fight against resistant fungal pathogens.

## Figures and Tables

**Table 1:** Minimal Inhibitory Concentration (MIC) values (in µg/mL) of RW3 against different species of fungi.

Compound	*C. albicans*	*C. auris*	*C. tropicalis*	*C. parapsilosis*	*P. kudravzevii*	*C. neoformans*	*C. gattii*	*S. brasiliensis*
RW3	31.3	15.6	125	125	15.6	3.9	7.8	15.6
Itraconazole	ND *	ND	ND	ND	ND	ND	ND	0.5
Fluconazole	0.5	64	0.25	1.0	32	2.0	2.0	ND
Caspofungin	ND	0.05	ND	ND	ND	ND	ND	ND

* ND = not determined.

**Table 2:** Final classification of RW3 according to its irritation potential in the HET-CAM test, German protocol.

Sample	Irritation Score	Classification
0.1 M NaOH	12.00	Severe irritant
NaCl 0.9%	0.00	Non-irritant
1%DMSO in saline	0.00	Non-irritant
RW3 (100 µM)	0.76	Non-irritant

**Table 3:** Experimentally determined solubility of RW3 at various pH values and 37 °C (n = 3).

pH	pH After Addition of RW3	Final pH After 24 h Shaking	Average Solubility mg/mL (±SD)
2.0	2.08	2.14	0.67 ± 0.001
4.5	4.52	4.61	1.01 ± 0.01
6.8	6.89	6.85	2.76 ± 0.09

**Table 4:** Permeability of RW3 in the Caco-2 cell model, in apical−basolateral (A-B) and basolateral−apical (B-A) directions (n = 3).

	Caco-2 Papp (×10^−6^ cm/s)
A-B	23.08 ± 9.69
B-A	17.68 ± 7.19
Ratio Efflux Papp B-A/Papp A-B	0.76

## Data Availability

The original contributions presented in this study are included in the article. Further inquiries can be directed to the corresponding author.

## Abbreviations

The following abbreviations are used in this manuscript:

ATCC	American Type Culture Collection
DMEM	Dulbecco’s Modified Eagle Medium
HET-CAM	Hen’s Egg Test on Chorioallantoic Membrane
MIC	Minimal Inhibitory Concentration
NMR	Nuclear Magnetic Resonance
TEER	Transepithelial Electrical Resistance
TLC	Thin-layer Cshromatography
TMS	Tetramethylsilane
